# The Evaluation of Phenolic Acids and Flavonoids Content and Antiprotozoal Activity of *Eryngium* Species Biomass Produced by Biotechnological Methods

**DOI:** 10.3390/molecules27020363

**Published:** 2022-01-07

**Authors:** Małgorzata Kikowska, Justyna Chanaj-Kaczmarek, Monika Derda, Anna Budzianowska, Barbara Thiem, Halina Ekiert, Agnieszka Szopa

**Affiliations:** 1 Laboratory of Pharmaceutical Biology and Biotechnology, Department and Division of Practical Cosmetology and Skin Diseases Prophylaxis, Collegium Pharmaceuticum, Poznan University of Medical Sciences, 3 Rokietnicka St., 60-806 Poznań, Poland; abudzian@ump.edu.pl (A.B.); bthiem@ump.edu.pl (B.T.); 2 Department of Pharmacognosy, Collegium Pharmaceuticum, Poznan University of Medical Sciences, 3 Rokietnicka St., 60-806 Poznań, Poland; justyna.chanaj-kaczmarek@ump.edu.pl; 3 Department of Biology and Medical Parasitology, Poznan University of Medical Sciences, 10 Fredry St., 61-701 Poznań, Poland; mderda@ump.edu.pl; 4 Department of Pharmaceutical Botany, Collegium Medicum, Jagiellonian University, 9 Medyczna St., 30-688 Kraków, Poland; halina.ekiert@uj.edu.pl (H.E.); a.szopa@uj.edu.pl (A.S.)

**Keywords:** eryngo, in vitro cultures, extracts, phenolic acids, flavonoids, acanthamoebic activity

## Abstract

Three species from the *Eryngium* L. genus—*E. campestre*, *E. maritimum*, and *E. planum*, plants with a rich chemical composition, were selected for phytochemical and biological studies. The applied biotechnological methods allowed to obtain the biomass of these rare or protected species in the form of multiplied shoots (stationary system) and roots cultured in a liquid medium (agitated system). In the extracts from the raw material obtained under in vitro conditions, the content of selected phenolic acids and flavonoids (HPLC-DAD method) as well as the total of polyphenols (Folin–Ciocalteu assay) were quantified. The highest amount of all phenolic compounds was found in extracts from *E. planum* roots (950.90 ± 33.52 mg/100 g d.w.), and the lowest from *E. campestre* roots (285.00 ± 10.07 mg/100 g d.w.). The quantitatively dominant compound proved to be rosmarinic acid. The highest amounts were confirmed for *E. planum* root extract (694.58 mg/100 g d.w.), followed by *E. planum* (388.95 mg/100 g d.w.) and *E. campestre* (325.85 mg/100 g d.w.) shoot extracts. The total content of polyphenols was always increased in the biomass from in vitro cultures in comparison to the analogous organs of intact plants of each species. The obtained extracts were assessed for antiprotozoal activity against *Acanthamoeba* sp. The strength of biological activity of the extracts correlated with the content of phenolic compounds. To our knowledge, this is the first report on the amoebicidal activity of *E. campestre*, *E. maritimum*, and *E. planum* extracts from biomass produced by biotechnological methods.

## 1. Introduction

*Eryngium* L. (Sea Holly, Eryngo) comprises about 230–250 species and is, therefore, the largest genus of the Saniculoideae subfamily from the Apiaceae family. This taxon is widespread in Central Asia, America, Central, and Southeast Europe, North Africa, and Australia [1,2]. Some of them, including *E. campestre*, *E. maritimum*, and *E. planum*, are described in Flora Europaea [3]. Among Polish taxa of the genus, *E. maritimum* is endangered and protected, *E. planum* and *E. campestre* are rare and can be found only in restricted regions [4].

Based on the review of the scientific literature up to now, the phytochemical investigations of those three *Eryngium* species revealed the presence of triterpenoid saponins, phenolic acids, flavonoids, coumarin derivatives, the essential oil, polyacetylenes, phytosterols, and ecdysteroids [4,5]. Some taxa have been used as ornamental, vegetable, and medicinal plants in folk medicine. These three species appearing in the Polish flora have been known from traditional medicine in Poland and Europe. The main raw materials — herbs (*Eryngii herba*) and roots (*Eryngii radix*) are used as an antitussive, in various types of urinary tract diseases such as kidney and bladder stones, nephrosis or other kidney problems, employed to cure wounds, burns, pains, and hemorrhoids, moreover, applied as a remedy for snake venoms and scorpion stings [6].

Limited generative reproduction affected by low seed production, low germination, high juvenile mortality as well as habitats exploitation causes deficiency in the availability of plant material from natural sites. Plant in vitro cultures of those rare and endangered species as well as biotechnological methods of application may provide biomass with the enhanced accumulation of desired secondary metabolites without collecting plants from natural sites. Under natural conditions, the biosynthesis of secondary metabolites in plants is slow and generally very little effective. Methods of plant cultures in vitro enable strict control and optimization of biosynthesis. The project intends to achieve a constant and renewable plant biomass with a metabolic profile similar to ground plants, aimed at higher accumulation of specific secondary metabolites [6].

Parasitic free-living amoebae of the *Acanthamoeba* genus are cosmopolitan organisms widespread in the world—they occur in soil and air, as well as in fresh and salt water, but also in swimming pools, fountains, smears under the showers, bottled water, and lens fluids. Under favorable conditions, these amoebae can become pathogenic organisms—they can be the etiological factor of granulomatous amoebic encephalitis, keratitis acanthamoeba, as well as a cause of changes in many organs of humans and animals [7,8]. They access organisms via the respiratory tract, wounds, lesions, or skin ulcerations. Due to the fact that the life cycle of free-living amoebae is relatively simple, and their isolation and axenization do not pose any major difficulties, it is possible to culture them in in vitro conditions and study the influence of compounds, fractions, or plant extracts on the antiprotozoal activity against *Acanthamoeba* sp. [7,8,9].

The aim of the present study was to evaluate the phenolic compounds content and to investigate the in vitro antiamoebic activity of the extracts obtained from biomass produced by biotechnological methods (shoot and root in vitro culture) of *E. campestre*, *E. maritimum*, and *E. planum* on the growth and development of trophozoites.

## 2. Results and Discussion

**Figure 1:**
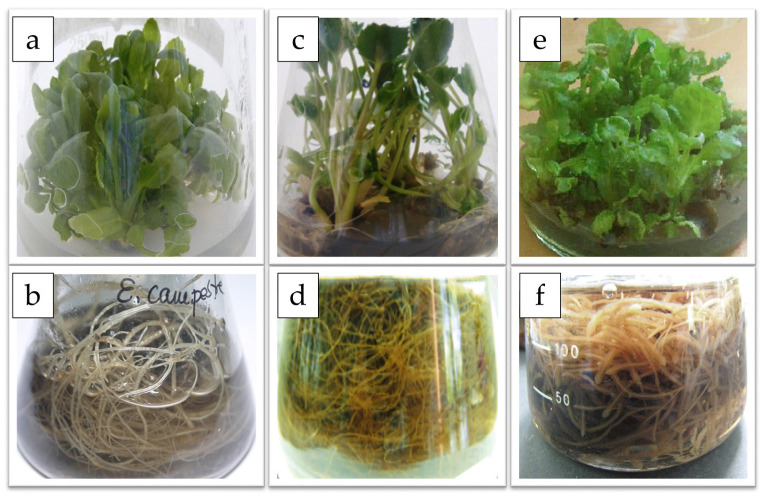
In vitro shoot and root cultures of: (**a**,**b**) *Eryngium campestre* L.; (**c**,**d**) *Eryngium maritimum* L.; (**e**,**f**) *Eryngium planum* L.

The introduction of selected *Eryngium* species (*E. campestre*, *E. maritimum*, and *E. planum*) into in vitro culture conditions was previously described [10,11,12]. Material for biotechnological, phytochemical, and biological research came from stabilized cultures of the described species. Briefly, initial shoot cultures originated from aseptic seedling explants (fruits obtained from plants from natural sites) of *E. campestre* and *E. planum,* or stem fragments with nodes (young plants collected from a botanical garden) of *E. maritimum*. Despite many attempts, it was not possible to obtain seedlings from axenic seeds of *E. maritimum* continuing their development in in vitro cultures. For the subsequent growth of shoot cultures and the regeneration of the largest number of shoots, the media were supplemented with plant growth regulators. Adventitious root cultures were initiated from explants derived from rooted plantlets, on a solid medium in the process of micropropagation (Figure 1).

Shoot multiplication by the development of axillary buds of three *Eryngium* species is a fast and effective strategy for obtaining a relatively large number of new shoots (Table 1). The studied species were characterized by a high shoot induction coefficient (96.3–100%). For these species, it was possible to obtain a high number of shoots (except for *E. maritimum*), which guarantees obtaining the necessary biomass for phytochemical and biological analyzes.

The shoots were characterized by the correct morphology and physiology, did not show hyperhydricity, necrosis, or callus formation at the shoot base, therefore, these raw materials can be described as homogeneous and of high quality (Figure 1).

The presence of cytokinin in the medium was an important factor in the process of shoot multiplication. According to the literature, 6-benzyladenine (whether used alone or in combination with auxin) influenced the number of micro-shoots formed in in vitro cultures. This relationship was also observed for other species belonging to the Apiaceae family: *Thapsia garganica* L. [13], *Arracacia xanthorrhiza* Bancr. [14], or *Anethum graveolens* L. [15].

Roots play an important role not only in plant growth and development but also constitute a place of accumulation of secondary metabolites, in large part also those useful from a pharmaceutical point of view. Moreover, the adventitious roots show significant productivity, which is why they are often an important source of these substances [16]. In this study, a method of obtaining and cultivating adventitious roots in liquid media was developed for the domestic species of *Eryngium*. The main pharmaceutical raw material *Eryngium* is the root of intact plants, rich in secondary metabolites, therefore, in vitro cultures of these organs were established.

In this experiment, fragments of roots regenerated in in vitro cultures of micropropagated plants were used to establish root lines agitated in liquid media (Figure 1). Approximately 2 cm long sections of main roots containing a growth tip with apical meristem were taken and grown in liquid MS media supplemented with selected auxin—indole-3-acetic acid (IAA). The adventitious roots gradually grew in length and the lateral root system developed. The roots of two species—*E. campestre* and *E. planum*—grew fastest; much worse—*E. maritimum* (Table 2). However, the fresh and dry weight gains were relatively small compared to other species.

Under the HPLC-DAD analyses in the studied methanolic extracts of in vitro *Eryngium* spp. Cultures, the qualitative differences were noted (Table 3). In *E. campestre*, eight phenolic acids were present: chlorogenic, ferulic, 3,4-dihydroxyphenylacetic, caffeic (not detected in roots), protocatechuic, rosmarinic, syringic, 4-feruloylquinic acid, and 4-dihydroxybenzoic acids, and 5 flavonoids: kaempferol, quercitrin (not detected in shoots), rutoside (not detected in roots), quercetin, and astragalin. In *E. maritimum* were present nine phenolic acids: chlorogenic, ferulic, 3,4-dihydroxyphenylacetic, caffeic (not detected in roots), protocatechuic, rosmarinic, syringic, vanillic, 4-feruloylquinic acid and 4-dihydroxybenzoic acids, and tree flavonoids: kaempferol, quercitrin and rutoside). In *E. planum*, nine phenolic acids were present: chlorogenic, isochlorogenic, ferulic, 3,4-dihydroxyphenylacetic, caffeic, protocatechuic, rosmarinic, syringic, vanillic (not detected in roots), and 4-feruloylquinic acid, and tree flavonoids: kaempferol, quercitrin, and astragalin. Most of these acids (except chlorogenic, caffeic, rosmarinic and ferulic acids) were for the first time detected and quantified in the raw materials of these species (Table 3).

Undoubtedly, rosmarinic acid (RA) was the phenolic acid found in the highest concentration in all of the tested shoot and root in vitro cultures of *Eryngium* spp. The richest source of RA, based on our estimations, were root (694.58 mg/100 g d.w.) and shoot (388.95 mg/100 g d.w.) cultures of *E. planum* (Table 3). Within all detected phenolic compounds, RA was quantitatively dominant also in *E. maritimum* (181.27 mg/100 g d.w.—root; 174.51 mg/100 g d.w.—shoot cultures) and in *E. campestre* (100.91 mg/100 g d.w.—root; 325.85 mg/100 g d.w.—shoot cultures) (Table 3). RA was found most notably also before in *Eryngium* species, especially in shoots and roots of intact plants and micropropagated in vitro plantlets: *E. campestre* [12], *E. maritimum* [11], and *E. planum* [10]. The qualitative screening analysis of *Eryngium* genus performed by the team of Le Claire revealed the presence of rosmarinic acid in the root extracts of 11 *Eryngium* species [17].

Chlorogenic acid and 3,4-dihydroxyphenylacetic acid were found in much lower concentrations than RA, but relatively higher than for other phenolic acids. Three conjugates of caffeic acid and quinic acid namely neochlorogenic acid (5-caffeoylquinic acid), chlorogenic acid (3-caffeoylquinic acid), and isochlorogenic acid (3,5-dicaffeoylquinic acid) were demonstrated: in shoots and roots obtained from ground plants and in vitro-propagated *E. campestre* [12,18], *E. maritimum* [11,18], and *E. planum* [10,18].

Comparing the total phenolic acids contents estimated with the HPLC method, *E. planum* root cultures proved to be the richest source of these compounds (924.21 mg/100 g d.w.), mainly due to the high content of RA (Table 3). Moreover, high phenolic acid total contents were detected for *E. campestre* (739.26 mg/100 g d.w.) and *E. planum* (729.10 mg/100 g d.w.) shoot cultures (Table 3).

The flavonoid estimations with HPLC showed also quantitative differences within individual compounds depending on studied species as well as *Eryngium* spp. (Table 3). Quantitative analyzes generally show that shoots from in vitro cultures have a higher content of flavonoids than roots grown under the same conditions. The main flavonoid for *E. campestre* was astragalin (57.05 mg/100 g d.w.—shoots, 6.21 mg/100 g d.w.—roots), for *E. maritimum*—rutoside (14.32 mg/100 g d.w.—shoots, 12.16 mg/100 g d.w.—roots) and for *E. planum* was quercitrin (11.25 mg/100 g d.w.—shoots, 14.41 mg/100 g d.w.—roots) (Table 3). Comparing the total contents of flavonoids in in vitro cultures of analogous organs of *Eryngium* spp., a trend according to the formula can be noticed: *E. campestre* > *E. maritimum* > *E. planum*. Summing up, the most valuable source of flavonoids is the in vitro shoots of *E. campestre*, mainly due to the very high content of astragalin (Table 3).

Flavonoids characterized in these species concern plants from their natural sites. The phytochemical study initiated by the team of Hiller and Leokadia in the 1980s revealed the presence of two derivatives of kaempferol in the aerial part of *E. planum* L. [19,20]. In more widely studied *E. campestre* L., the following compounds were identified: quercitrin, isoquercitrin, rutin, luteolin 7-*O*-β-d-glucopyranoside [21], kaempferol 3-*O*-β-d-(2′-*p*-*E*-hydroxycinnamoyl)-glucopyranoside, and kaempferol 3-*O*-β-d-(2′-*p*-*Z*-hydroxycinnamoyl)-glucopyranoside [22]. These findings encourage the investigation of the aerial part of *E. campestre* l. and in consequence 11 flavonol compounds were isolated. Three quercetin glycosides, five isorhamnetin glycosides, and three myricetin glycosides were elucidated by spectroscopic and chemical methods employing UV-visible spectrophotometer, MS, and NMR spectrometer [23]. *E. maritimum* L. contains mainly astragalin, 3-β-d-glucopyranoside7-*O*-l-rhamnopyranoside, kaempferol, and isoquercitrin [24]. Polyphenolic compounds identified by Conea et al. in the *Eryngium* (*E. planum* L., *E. campestre* L., and *E. maritimum* L.) tinctures were isoquercitrin, quercitrin, rutoside, and kaempferol [18]. Through the multiplication of *Eryngium* biomass in vitro, it became possible to supplement the knowledge on the presence and content of phenolic compounds in the raw materials of these species.

Based on the comparison of total phenolic compounds contents estimated with the HPLC method, the richest source of these compounds was root cultures of *E. planum* (950.90 mg/100 g d.w.) and shoot cultures of *E. campestre* (846.27 mg/100 g d.w.) (Table 3).

Quantitative studies of the content of polyphenols using spectrophotometric methods according to the modified method of Meng et al. [25] with the use of the Folin–Ciocalteu reagent were carried out comparatively for the raw materials of intact plants and organs (shoots and roots) from in vitro cultures of three *Eryngium* species (Table 4).

The content of the sum of polyphenols in the tested raw materials was always higher in the biomass from in vitro cultures than in the analogous organs from intact plants for selected species (Table 4). In particular, the root biomass from in vitro cultures was characterized by a much higher polyphenol content than the roots of intact plants. The highest content of total polyphenols (2254 of gallic acid equivalent (mg/100 g)) was found in the roots of in vitro cultures of *E. campestre*, which is consistent with the observation of their morphology—these were the youngest roots, characterized by a large number of lateral roots.

The antiprotozoal action of the extracts obtained from *Eryngium*, both from in vitro shoot and root cultures, was evaluated in vitro against *Acanthamoeba* sp. strain Ac55. The results of the study indicated that the extracts inhibited the growth of trophozoites to varying degrees. The dependence of the effect on the extract concentration and treatment time was noted (Table 5, Table 6, Table 7 and Table 8).

In the case of *E. campestre*, the strongest effect was observed for in vitro shoot culture (Table 5). The higher sample concentration was applied, the more effective activity was observed in comparison with the control during the same time interval. It can be noticed that the shoot extract activity was potent from the beginning of the treatment and remained at a similar level (5.0 mg/mL) or a significantly lower level (0.5 mg/L and 2.5 mg/L) during the next days, while the activity of the root extract increased with the time of treatment (0.5 mg/L and 2.5 mg/L).

In the case of *E. maritimum*, a stronger effect was observed for in vitro root culture (Table 6). Generally, the higher sample concentration was applied, the more effective activity was observed in comparison with the control during the same time interval. It can be noticed that the activity of both shoot and root extracts (0.5 mg/L and 2.5 mg/L) increased with the time of treatment, while the activity of the extract of a higher concentration (5.0 mg/L) remained at a statistically similar level.

In the case of *E. planum*, a strong effect was observed both for in vitro shoot and root culture (Table 7). Generally, no statistical differences were observed in the activity of the extracts between the higher (2.5 mg/L) and the highest concentration (5.0 mg/L) during the same time interval. Moreover, no statistical differences were observed in the activity of the extracts between the 3rd and 4th day taking into consideration the highest concentrations of the extract. On the fourth day of exposure of trophozoites to the shoot culture extract, regardless of the concentration, cysts appeared.

The lowest IC_50_ index was calculated for the *E. planum* shoot culture extract. On the second day of *Acanthamoeba* trophozoites treatment, the IC_50_ value was 0.25 mg/mL. During the entire treatment period, the lowest IC_50_ was obtained for the extract of *E. campestre* shoot culture and shoot and root cultures of *E. planum* (Table 8).

**Figure 2:**
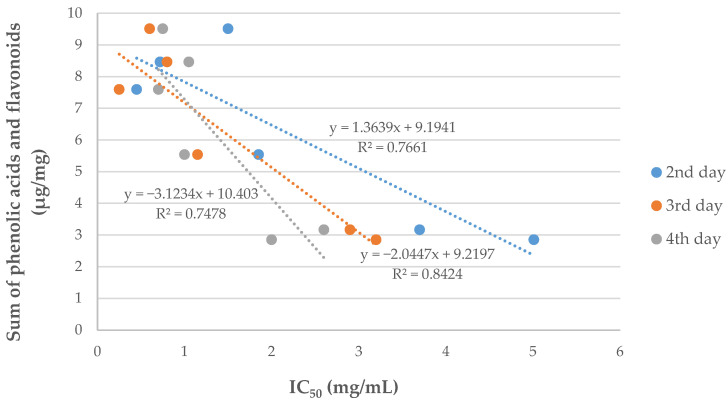
Correlation between IC_50_ values and the sum of phenolic acids and flavonoids determined by HPLC method in *Eryngium* species.

The obtained extracts were assessed for antiprotozoal activity against *Acanthamoeba* sp. The strength of biological activity of the extracts correlated with the content of phenolic compounds. The highest amounts of phenolic acids and flavonoids were confirmed for *E. planum* root extract, followed by *E. planum* and *E. campestre* shoot extracts, which was correlated with the results of IC_50_ for those extracts. The conducted studies showed a strong correlation between the activity of *Eryngium* species extracts on *Acanthamoeba* trophozoites proliferation with the sum of flavonoids and phenolic acids determined by using the HPLC-DAD method (R^2^ in the range 0.7478–0.8424) (Figure 2). This effect of the synergistic action of phenolic compounds may be due to the presence in the plant materials of chlorogenic acid [26], rosmarinic acid [27], and quercetin [28], which are well known as amoebicidal agents.

Previously, the evaluation of the amoebicidal activity of *E. planum* was conducted for the extracts and fractions obtained from leaves and roots of intact plants. Among different fractions from leaf and root ethanolic extracts: flavonoid, flavonoid-saponin, and saponin assayed for the antiamoebic activity studies, the phenolic acid fraction from roots at the concentration of 5.0 mg/L showed the activity on the *A. castellanii* trophozoites. According to the authors, this activity is correlated with the antioxidant activity of phenolic acids. This class of phenolic compounds may cause damage to the plasma membrane, which results in a leakage of intracellular constituents from the cell [29]. For the alpine species (*Eryngium alpinum*), the activity of the extract from the leaves of intact plants and in vitro shoot cultures was compared. The extract of shoots multiplied in vitro showed the highest antiamoebicidal effect already on the second day of treatment: the percent of inhibition of trophozoites was 81.14%, 66.38%, and 54.99% at the concentrations of 5 mg/mL, 2.5 mg/mL, and 0.5 mg/mL, respectively. The extract from shoots of intact plants at a dose of 0.5 and 2.5 mg/mL weakly inhibited the development of trophozoites [30].

In our experiment, on the fourth day of exposure of trophozoites to the *E. planum* shoot culture extract, regardless of the concentration, cysts appeared. The parasite has a strong ability to transform into a dormant cyst stage under stressful conditions [8]. Unfortunately, the cyst walls provide a physical barrier for drugs/extracts/fraction or isolated compounds to target amoeba residing within the shell [31]. In the authors opinion, the discovery of various antiacanthamoebic natural products tested in vitro has not been able to enter the drug development process due to their inefficacy against the cysts.

In the literature on the subject, more scientific information on the plant extracts with the amoebicidal or amoebistatic activity against pathogenic strains of *Acanthamoeba* spp. can be found regarding the extracts from aerial parts of *Centaurea bella*, *C. daghestanica*, *Rhaponticum pulchrum*, *Tanacetum vulgare* [32], roots and leaves of *Rubus chamaemorus*, *Pueraria lobata*, *Solidago* spp. [33], leaves and calluses of *Passiflora* spp. [34], calluses, leaves, and roots of micropropagated plantlets of *Chaenomeles japonica* [35]. *Buddleia cordata* is a plant of potential and practical use in the treatment of acanthamoebosis—the therapy uses extracts from the roots, bark, and fruits of this plant. It was noted that the chemically active substance in these extracts is linarin, which belongs to the flavones [36]. Flavonoids and phenolic acids are the phenolic compounds that may be found to exert a strong antiprotozoal effect [26,27,28,37]. The problem of treating acanthamoebiasis with the use of substances of plant origin has recently become very popular—the therapeutic properties of other plants are still being investigated.

To our knowledge, this is the first report on the amoebicidal activity of *E. campestre*, *E. maritimum*, and *E. planum* extracts from biomass produced by biotechnological methods.

## 3. Materials and Methods

### 3.1. Plant Material

Fruits of *E. campestre* were collected from steppe reserve Owczary (Sękowa, Poland) and of *E. planum* from natural habitats in Poland (Lukaszewo, Kuyavian-Pomeranian province). Primary explants—shoot fragments with lateral buds of. *E. maritimum*—were shared by the Botanical Garden of Adam Mickiewicz University in Poznań (Poland). The appropriate explants were surface disinfected and introduced to the in vitro conditions according to the procedure adopted by Thiem et al. [10] and Kikowska et al. [11,12].

### 3.2. Shoot Cultures

Shoots were multiplied through an axillary branching method by repetitive transfer of suitable explant. Multishoots were divided into single shoots and transferred to new medium solidified with 7.6 g/L agar with the same supplementation every 5–6 weeks. *E. campestre*, *E. planum*, and *E. maritimum* were multiplied on MS medium [38] enriched with 6-benzyladenine (BA, 1.0 mg/L) and indole-3-acetic acid (IAA, 1.0 mg/L). The percentage of explants that proliferated buds, total number of shoots per explant, and length of shoots were recorded after 6 weeks of 18–19th subculture. Multiplication of shoots was replicated three times at least with 10 explants per treatment. The shoots were multiplied according to the procedure adopted by Thiem et al. [10] and Kikowska et al. [11,12].

### 3.3. Root Cultures

Root fragments with tips (1.0–2.0 cm long) obtained from axenic plantlets were used for adventitious root cultures initiation. The explants were transferred into MS liquid media with an auxin IAA (1.0 mg/L). The cultures were maintained on a rotary shaker at 100 rpm. Root cultures were inoculated into the same liquid media and the same culture conditions as the one employed for routine subculturing were applied. The root cultures were subcultured at 5-week intervals. The roots were maintained according to the procedure adopted by Thiem et al. [10] and Kikowska et al. [11,12].

### 3.4. Culture Conditions

The shoot cultures were grown under artificial light—55 μmol/m^2^s (16 h light/8 h dark photoperiod) and the root cultures were cultured in the darkness. Both cultures were maintained at a temperature of 21 °C ± 2 °C.

### 3.5. HPLC–DAD Analysis

For chromatographic analyses, the fresh biomass of in vitro shoots and roots of *Eryngium* species (*E. campestre*, *E. maritimum*, *E. planum*), was dried at 40 °C for 24 h to a constant weight. For extract preparation, dried and pulverized material samples, 0.5 g dry weight (DW) each, were extracted by sonication (Polsonic**^®^** 3, Warsaw, Poland) in methanol (5 mL) two times for 30 min. The validated HPLC–DAD method was employed for the analyses of phenolic acids and flavonoids in the extracts (according to Ellnain-Wojtaszek and Zgorka [39] and Szopa et al. [40]). The HPLC–DAD apparatus and conditions were profiled by us previously [40,41] The recognition of compounds was carried out on the comparison of UV spectra (λ = 200–400 nm) and retention time of the reference substances. Moreover, the internal standard method was used. The quantification of compounds was performed by peak measurements and the standard curves method. The reference compounds were bought from Sigma-Aldrich (Saint Louis, MO, USA) (phenolic acids: 3,4-dihydroxyphenylacetic acid, caftaric acid, caffeic acid, chlorogenic acid, 2-coumaric, 3-coumaric, 4-coumaric acids, ferulic acid, 4-feruloylquinic acid, gallic acid, gentisic acid, hydrocaffeic acid, 4-dihydroxybenzoic, 4-hydroxybenzoic acid, isochlorogenic acid, isoferulic acid, neochlorogenic acid, protocatechuic acid, rosmarinic acid, salicylic acid, sinapic acid, syringic acid and vanillic acid, and benzoic and cinnamic acids (precursors of phenolic acids); and flavonoids—aglycones: isorhamnetin kaempferol, luteolin, quercetin, rhamnetin, and myricetin and glycosides: apigetrin, astragalin cynaroside, hyperoside, populnin, quercitrin, rutoside, trifolin, and vitexin).

### 3.6. Total Phenolics Content

The content of the sum of polyphenols was determined with the Folin–Ciocalteu (Sigma-Aldrich, Saint Louis, MO, USA) reagent according to the modified method of Meng et al. [25]. The dried and weighed plant material (shoots and roots from in vitro cultures and intact plants) was extracted four times in 70% (*v*/*v*) MeOH in a water bath, under reflux, at a temperature of 95 °C, each time for 1 h. The obtained extracts are combined and concentrated to a volume of 25 mL on a vacuum evaporator at 40 °C. Briefly, 0.1 mL of each extract or gallic acid solution at different concentrations (0.02–0.08 mg/mL) was mixed with 0.1 mL of Folin–Ciocalteu reagent and allowed to react at room temperature for 3 min. Finally, 1.0 mL of aqueous solution of sodium carbonate (7.0%, *w*/*v*) was added, and the mixture was incubated in the dark at room temperature for 60 min. The blank sample contained water instead of the extract or gallic acid solution. From each extract, three samples were prepared, from each four analyzes were performed. The measurements were made at the wavelength λ = 760 nm, in the Perkin-Elmer Lambda (Norwalk, CT, USA) 35 UV/VIS apparatus. Gallic acid (GA) was used as an external standard to plot the calibration curve (y = 94.065x + 0.0331, R² = 0.9999) and the results were expressed as milligrams of GA equivalent per gram of dry weight of the plant material. The values were expressed as the mean of six replications ± SD.

### 3.7. Antiamoebic Activity

In this study, the *Acanthamoeba* sp. strain Ac55 (isolated from a patient with keratitis, T4 genotype) deposited in GenBank (NCBI) under accession number KP120880 was used. The amoebae were axenically cultured on a liquid medium containing 2% Bacto-Casitone. The methanolic extracts were dissolved in 50 µL of dimethylsulfoxide (DMSO) and then diluted with distilled water to obtain the appropriate concentrations. These dilutions were added to the axenic culture of amoebae containing 5 × 10^4^ cells/mL at the concentrations of 0.5–5 mg/mL. The increase or decrease in the number of amoebae was checked at 24 h intervals for three days (2nd, 3rd, 4th) in a Thoma hemocytometer chamber. The control consisted of cultured trophozoites without extracts. The relationship between fraction concentration and the time of treatment of trophozoite cultures was investigated. The method was described by us previously [29,30,31,32,33,34].

### 3.8. Statistical Analysis

The data from biotechnological, phytochemical, and biological experiments were analyzed using a one-way analysis of variance (ANOVA) and the statistical significance was determined using Duncan’s post hoc test (*p*-value < 0.05). All the analyses were conducted employing STATISTICA v. 13 (StatSoft, Inc. 2015, Kraków, Poland).

## 4. Conclusions

Shoot and root in vitro cultures of *Eryngium* species may be considered a valuable alternative source of biomass that is rich in valuable bioactive compounds such as phenolic acids and flavonoids. The content of the sum of polyphenols in *Eryngium* species was always higher in the biomass from in vitro cultures than in the analogous organs of ground plants of each species. The strength of antiprotozoal activity of extracts correlated with the content of phenolic compounds determined in them. The results suggest that the extracts from *Eryngium* spp. may be promising candidates for *Acanthamoeba* treatment.

## Figures and Tables

**Table 1:** The growth parameters of stabilized in vitro shoot cultures of *Eryngium* species.

Species Studied	Explants that Proliferated Buds (%)	Shoot Number per Explant (SE)	Shoot Length (cm SE)	Leaf Number Per Shoot (SE)
*E. campestre*	100	11.34 ± 0.46 ^b^	3.56 ± 0.12 ^a^	5.15 ± 0.20 ^a^
*E. maritimum*	96.3	4.1 ± 0.30 ^c^	3.1 ± 0.03 ^b^	3.90 ± 0.07 ^a^
*E. planum*	100	14.56 ± 0.20 ^a^	4.52 ± 0.04 ^a^	5.29 ± 0.04 ^a^

The results are presented as the mean ± SE of three independent experiments with 30 explants per treatment. Mean values within a column with the same letter are not significantly different at *p* < 0.05 using Duncan’s multiple range test. The first letter of the alphabet for the highest values, the next for statistically significant decreasing values.

**Table 2:** The root biomass (g ± SE) of *Eryngium* spp. in MS liquid medium after 24 weeks of in vitro culture.

Species Studied	Fresh Weight [g]	Dried Weight [g]
*E. campestre*	27.1 ± 0.60 ^a^	5.8 ± 0.21 ^a^
*E. maritimum*	16.4 ± 0.78 ^b^	4.1 ± 0.43 ^b^
*E. planum*	25.1 ± 1.45 ^a^	5.8 ± 0.16 ^a^

The results are presented as the mean ± SE of three independent experiments with 5 explants per treatment. Mean values within a column with the same letter are not significantly different at *p* < 0.05 using Duncan’s multiple range test. The first letter of the alphabet for the highest values, the next for statistically significant decreasing values.

**Table 3:** The contents (mg/100 g d.w. ± SD) of estimated phenolic acids and flavonoids in the shoot and root in vitro cultures of different *Eryngium* species.

Compounds	*E. campestre*		*E. maritimum*		*E. planum*	
	Shoot Culture	Root Culture	Shoot Culture	Root Culture	Shoot Culture	Root Culture
Phenolic acid						
Chlorogenic acid	154.47 ± 5.44 ^a^	13.20 ± 0.47 ^e^	30.51 ± 1.08 ^d^	140.56 ± 4.95 ^b^	107.91 ± 3.80 ^c^	99.67 ± 3.51 ^c^
Isochlorogenic acid	n.d.	n.d	n.d.	n.d.	46.30 ± 1.63 ^a^	17.34 ± 0.61 ^b^
Ferulic acid	23.25± 0.82 ^b^	9.35 ± 0.33 ^b^	7.76 ± 0.27 ^d^	8.33 ± 0.29 ^c^	3.17 ± 0.11 ^f^	3.51 ± 0.12 ^e^
3,4-Dihydroxyphenylacetic acid	88.88 ± 3.13 ^b^	50.91 ± 1.79 ^c^	40.76 ± 1.44 ^d^	108.92 ± 3.84 ^a^	104.05 ± 3.67 ^a^	52.21 ± 1.84 ^c^
Caffeic acid	4.03 ± 0.14 ^e^	n.d.	4.77 ± 0.17 ^d^	8.81 ± 0.31 ^c^	16.51 ± 0.58 ^a^	14.78± 0.52 ^b^
Protocatechuic acid	8.12 ± 0.29 ^a^	1.81 ± 0.06 ^e^	3.61 ± 0.13 ^d^	7.41 ± 0.26 ^b^	8.57 ± 0.30 ^a^	4.07 ± 0.14 ^c^
Rosmarinic acid (RA)	325.85 ± 11.48 ^c^	100.91 ± 3.56 ^e^	174.51 ± 6.15 ^d^	181.27 ± 6.39 ^d^	388.95 ± 13.70 ^b^	694.58 ± 24.47 ^a^
Syringic acid	0.99 ± 0.04 ^c^	5.45 ± 0.19 ^a^	1.87 ± 0.07 ^b^	1.86 ± 0.07 ^b^	0.37 ± 0.01 ^e^	0.88 ± 0.03 ^d^
Vanillic acid	n.d.	n.d.	2.54 ± 0.09 ^b^	2.34 ± 0.08 ^c^	5.68 ± 0.20 ^a^	n.d.
4-Feruloylquinic acid	9.83 ± 0.35 ^d^	46.90 ± 1.65 ^b^	8.44 ± 0.30 ^e^	64.96 ± 2.29 ^a^	47.59 ± 1.68 ^b^	38.17 ± 1.34 ^c^
4-Dihydroxybenzoic acid	122.84 ± 4.33 ^a^	n.d.	7.56 ± 0.27 ^b^	n.d.	n.d.	n.d.
Sum of phenolic acids	738.26 ± 26.02 ^b^	228.53 ± 8.05 ^e^	282.33 ± 9.90 ^d^	524.46 ± 20.77 ^c^	729.10 ± 25.68 ^b^	925.21 ± 32.58 ^a^
Flavonoids						
Kaempferol	14.53 ± 0.51 ^a^	5.85 ± 0.20 ^e^	13.10 ± 0.46 ^b^	6.41 ± 0.22 ^d^	3.71 ± 0.13 ^f^	8.82 ± 0.31 ^c^
Quercitrin	n.d.	21.45 ± 0.75 ^a^	6.95 ± 0.24 ^d^	10.55 ± 0.37 ^c^	11.25 ± 0.40 ^c^	14.41 ± 0.51 ^b^
Rutoside	29.51 ± 1.04 ^a^	n.d.	14.32 ± 0.50 ^b^	12.16 ± 0.43 ^c^	n.d.	n.d.
Quercetin	6.92 ± 0.24 ^b^	22.96 ± 0.81 ^a^	n.d.	n.d.	n.d.	n.d.
Astragalin	57.05 ± 1.44 ^a^	6.21 ± 0.26 ^c^	n.d.	n.d.	15.49 ± 0.63 ^b^	2.46 ± 0.12 ^d^
Sum of flavonoids	108.01 ± 3.23 ^a^	56.47 ± 2.02 ^b^	34.37 ± 1.20 ^c^	29.12 ± 1.02 ^d^	30.45 ± 1.16 ^d^	25.69 ± 0.94 ^e^
Sum of phenolic acids and flavonoids	846.27 ± 29.25 ^b^	285.00 ± 10.07 ^f^	316.70 ± 11.10 ^e^	553.65 ± 21.79 ^d^	759.55 ± 26.84 ^c^	950.90 ± 33.52 ^a^

n.d.—not detected. Mean values within a raw with the same letter are not significantly different at *p* < 0.05 using Duncan’s multiple range test. The first letter of the alphabet for the highest values, the next for statistically significant decreasing values.

**Table 4:** Total phenolic content in *Eryngium* species dry plant material evaluated by Folin–Ciocalteau method, expressed as gallic acid equivalent (mg/100 g).

Species Studied	Plant Origin	Organ	Polyphenols Content (mg GAE/100 g)
*E. campestre*	Intact plant	Shoots	945 ± 4 ^f^
	In vitro culture	Shoots	1507 ± 11 ^b^
	Intact plant	Roots	1030 ± 20 ^e^
	In vitro culture	Roots	2254 ± 10 ^a^
*E. maritimum*	Intact plant	Shoots	1120 ± 17 ^d^
	In vitro culture	Shoots	1551 ± 17 ^b^
	Intact plant	Roots	150 ± 1 ^h^
	In vitro culture	Roots	1179 ± 4 ^d^
*E. planum*	Intact plant	Shoots	1221 ± 12 ^c^
	In vitro culture	Shoots	1584 ± 18 ^b^
	Intact plant	Roots	245 ± 1 ^g^
	In vitro culture	Roots	1507 ± 11 ^b^

The results are presented as the mean ± SD of six independent repetitions. Mean values within a column with the same letter are not significantly different at *p* < 0.05 using Duncan’s multiple range test. The first letter of the alphabet for the highest values, the next for statistically significant decreasing values.

**Table 5:** Effect of extract from *Eryngium campestre* shoot and root in vitro cultures on inhibition of *Acanthamoeba* trophozoites during four days treatment.

Extracts Concentration	Duration of Treatment [days]					
	2nd Day		3rd Day		4th Day	
Shoot culture	MN ± SD	GI [%]	MN ± SD	GI [%]	MN ± SD	GI [%]
Control	10.11 ± 2.05 ^a,C^	0	15.72 ± 3.36 ^a,B^	0	38.39 ± 7.77 ^a,A^	0
0.5 mg/mL	5.81 ± 2.07 ^b,B^	42.54	9.06 ± 2.09 ^b,B^	42.37	23.88 ± 6.49 ^b,A^	37.80
2.5 mg/mL	1.71 ± 1.18 ^c,B^	83.09	4.33 ± 1.63 ^c,B^	72.46	10.75 ± 3.47 ^d,A^	72.00
5.0 mg/mL	0.72 ± 0.65 ^d,A^	92.88	1.11 ± 0.81 ^d,A^	92.94	1.41 ± 1.15 ^e,A^	96.33
Root culture	MN ± SD	GI [%]	MN ± SD	GI [%]	MN ± SD	GI [%]
Control	11.11 ± 1.85 ^a,C^	0	20.33 ± 3.80 ^a,B^	0	27.81 ± 3.64 ^a,A^	0
0.5 mg/mL	10.28 ± 2.84 ^ab,B^	7.48	15.50 ± 2.91 ^ab,AB^	23.76	20.5 ± 3.80 ^a,A^	27.55
2.5 mg/mL	7.78 ± 2.25 ^ab,B^	29.98	11.42 ± 3.07 ^b,AB^	43.83	12.44 ± 3.28 ^b,A^	55.27
5.0 mg/mL	6.23 ± 2.28 ^b,A^	43.93	7.20 ± 2.14 ^b,A^	64.59	7.93 ± 5.66 ^b,A^	71.49

Mean values within a raw (capital letters) and a column (small letters) with the same letter are not significantly different at *p* < 0.05 using Duncan’s multiple range test. The first letter of the alphabet for the highest values, the next for statistically significant decreasing values. *n* = 18. MN—mean number of trophozoites; GI—growth inhibition.

**Table 6:** Effect of extract from *Eryngium maritimum* shoot and root in vitro culture on inhibition of *Acanthamoeba* trophozoites during four days treatment.

Extracts Concentration	Duration of Treatment [days]					
	2nd Day		3rd Day		4th Day	
Shoot culture	MN ± SD	GI [%]	MN ± SD	GI [%]	MN ± SD	GI [%]
Control	5.89 ± 2.71 ^a,C^	0	19.72 ± 3.40 ^a,B^	0	27.22 ± 4.59 ^a,A^	0
0.5 mg/mL	4.53 ± 2.63 ^ab,C^	23.09	11.02 ± 1.71 ^b,B^	34.49	17.40 ± 4.17 ^b,A^	36.05
2.5 mg/mL	3.72 ± 1.59 ^ab,C^	36.85	7.08 ± 0.86 ^c,B^	44.10	14.00 ± 2.28 ^b,A^	48.57
5.0 mg/mL	2.00 ± 1.15 ^b,A^	66.05	2.17 ± 1.57 ^d,A^	89.00	2.27 ± 1.62 ^c,A^	91.67
Root culture	MN ± SD	GI [%]	MN ± SD	GI [%]	MN ± SD	GI [%]
control	5.89 ± 2.71 ^a,B^	0	9.72 ± 3.40 ^a,B^	0	17.22 ± 4.09 ^a,A^	0
0.5 mg/mL	4.45 ± 1.28 ^a,B^	24.32	7.44 ± 3.04 ^a,AB^	23.46	11.88 ± 4.01 ^a,A^	31.02
2.5 mg/mL	2.38 ± 0.38 ^b,B^	59.60	1.00 ± 0.50 ^b,A^	89.72	2.99 ± 2.45 ^b,A^	92.61
5.0 mg/mL	0.61 ± 0.45 ^c,A^	89.65	0.38 ± 0.06 ^c,A^	96.10	0.61 ± 0.39 ^b,A^	96.46

Mean values within a raw (capital letters) and a column (small letters) with the same letter are not significantly different at *p* < 0.05 using Duncan’s multiple range test. The first letter of the alphabet for the highest values, the next for statistically significant decreasing values. *n* = 18. MN—mean number of trophozoites; GI—growth inhibition.

**Table 7:** Effect of extract from *Eryngium planum* shoot and root in vitro culture on inhibition of *Acanthamoeba* trophozoites during four days treatment.

Extracts Concentration	Duration of Treatment [days]					
	2nd Day		3rd Day		4th Day	
Shoot culture	MN ± SD	GI [%]	MN ± SD	GI [%]	MN ± SD	GI [%]
Control	10.50 ± 1.83 ^a,B^	0	20.67 ± 3.37 ^a,A^	0	25.12 ± 4.34 ^a,A^	0
0.5 mg/mL	4.00 ± 2.57 ^b,B^	61.91	10.92 ± 3.00 ^b,A^	47.17	13.80 ± 4.55 ^b,A^	46.07
2.5 mg/mL	3.39 ± 2.31 ^b,A^	67.72	4.00 ± 2.31 ^c,A^	80.65	4.33 ± 1.93 ^c,A^	82.77
5.0 mg/mL	2.35 ± 1.35 ^b,A^	77.62	1.56 ± 1.30 ^c,A^	92.02	1.50 ± 1.17 ^c,A^	94.03
Root culture	MN ± SD	GI [%]	MN ± SD	GI [%]	MN ± SD	GI [%]
Control	9.18 ± 2.26 ^a,C^	0	21.89 ± 4.15 ^a,B^	0	32.89 ± 4.14 ^a,A^	0
0.5 mg/mL	6.06 ± 1.95 ^a,B^	33.99	11.47 ± 4.26 ^b,AB^	47.61	18.33 ± 2.62 ^b,A^	44.27
2.5 mg/mL	3.68 ± 1.03 ^b,B^	59.92	4.35 ± 2.35 ^c,B^	80.13	10.83 ± 2.80 ^c,A^	67.08
5.0 mg/mL	2.11 ± 1.59 ^b,A^	77.02	1.57 ± 1.06 ^c,A^	92.83	4.41 ± 2.30 ^d,A^	86.61

Mean values within a raw (capital letters) and a column (small letters) with the same letter are not significantly different at *p* < 0.05 using Duncan’s multiple range test. The first letter of the alphabet for the highest values, the next for statistically significant decreasing values. *n* = 18. MN—mean number of trophozoites; GI—growth inhibition.

**Table 8:** Determination of IC_50_ [mg/mL] for the studied extracts of *Eryngium* extracts on *Acanthamoeba* trophozoites proliferation in the culture medium.

*Eryngium* Species	In Vitro Culture	IC50 2nd Day [mg/mL]	IC50 3rd Day [mg/mL]	IC50 4th Day [mg/mL]
*E. campestre*	shoot culture	0.72	0.80	1.05
	root culture	>5.00	3.20	2.00
*E. maritimum*	shoot culture	3.70	2.90	2.60
	root culture	1.85	1.15	1.00
*E. planum*	shoot culture	0.45	0.25	0.70
	root culture	1.50	0.60	0.75

## Data Availability

The data presented in this study are available from the authors.

## Sample Availability

Samples of the biomass/extracts are available from the authors.
